# Correction: One health at the last mile: Multi-scale predictors of *Schistosoma japonicum* infection in southwest china across two decades of control

**DOI:** 10.1371/journal.pntd.0014273

**Published:** 2026-04-28

**Authors:** The PLOS Neglected Tropical Diseases Staff

## Notice of Republication

This article was republished on April 16^th^, 2026, to correct an error in the title: china was erroneously not capitalized. Please download this article again to view the correct version. The originally published, uncorrected article and the republished, corrected articles are provided here for reference.

## Supporting information

S1 File Originally published, uncorrected article. (PDF)

S2 File Republished, corrected article. (PDF)
