# Supplementary material for: One health at the last mile: Multi-scale predictors of Schistosoma japonicum infection in southwest China across two decades of control
Source: PLoS Negl Trop Dis. 2026 Feb 23;20(2):e0013573. doi: 10.1371/journal.pntd.0013573 (PMC12928498; doi:10.1371/journal.pntd.0013573)
Supplement: S1 File — A comprehensive description of our sampling strategy. (PDF) [file pntd.0013573.s001.pdf]

S1 File. Sampling Strategy. A comprehensive description of our sampling strategy.

Sampling Strategy

In 2007 we selected 53 villages from three counties in Sichuan where schistosomiasis had reemerged. A magnitude 7.9 earthquake in 2008 impacted one of the three study counties, leading us to discontinue research in this county. Observations from this county were not included in any of our models. In 2010, the 36 villages in the remaining two counties were surveyed. In 2016, surveillance records were reexamined to focus surveys on the highest-risk locations within the two counties, as schistosomiasis infections were declining. A total of 10 villages were selected for the 2016 survey, including 7 from the 2007 and 2010 surveys, and 3 that were newly added. In 2019, we surveyed 25 villages in the two counties including 9 of the villages surveyed in 2016, and 16 villages where surveillance records indicated possible ongoing or recent schistosomiasis transmission (4 of the these were surveyed in 2007 and 2010). In each village and timepoint, we conducted a panel survey, including a census of all residents aged 6 and older, collection of demographic data, recruiting these individuals for *S. japonicum* infection testing, and conducting household surveys.
