# Supplementary material for: One health at the last mile: Multi-scale predictors of Schistosoma japonicum infection in southwest China across two decades of control
Source: PLoS Negl Trop Dis. 2026 Feb 23;20(2):e0013573. doi: 10.1371/journal.pntd.0013573 (PMC12928498; doi:10.1371/journal.pntd.0013573)
Supplement: S1 Table — (PDF) [file pntd.0013573.s002.pdf]

S1 Table. Summary of predictors included in the analysis

Predictor	Detailed description (units)	Scale	Category	Variable Type	Notes
Rice area (H)	Area of rice cultivated by the household in the past year (mu)	Household	Agriculture	Continuous	Almost all rice is cultivated in the summer season.
Rice area (V)	Mean area of rice cultivated per household (mu)	Village	Agriculture	Continuous	
Dry crop area (H)	Total area of dry crops cultivated by the household in the past year (mu)	Household	Agriculture	Continuous	Most common dry crops are corn in the summer months, wheat and rapeseed in the winter months. Various vegetables are grown in both winter and summer seasons.
Dry crop area (V)	Mean area of dry crops cultivated per household (mu)	Village	Agriculture	Continuous	
Night soil rice (H)	Quantity of night soil applied to rice crops (buckets)	Household	Agriculture	Continuous	
Night soil rice (V)	Mean volume of night soil applied to rice crops per household (buckets)	Village	Agriculture	Continuous	This represents the frequency of night soil x the buckets of night soil used for each instance.
Night soil dry crops (H)	Quantity of night soil applied to dry crops (buckets)	Household	Agriculture	Continuous	See explanation for *Night Soil Rice (H)*
Night soil dry crops (V)	Mean volume of night soil applied to dry crops per household (buckets)	Village	Agriculture	Continuous	
Bovines (H)	Number of bovines owned by household	Household	Animal Reservoir	Continuous	Surveys allowed respondents to input any integer value of water buffalo and cows they owned.
Bovines (V)	Percent of households that own bovines	Village	Animal Reservoir	Continuous	
Cats (H)	Household owns cats	Household	Animal Reservoir	Binary	
Cats (V)	Percent of households that own cats	Village	Animal Reservoir	Continuous	

Predictor	Detailed description (units)	Scale	Category	Variable Type	Notes
Dogs (H)	Household owns dogs	Household	Animal Reservoir	Binary	
Dogs (V)	Percent of households that own dogs	Village	Animal Reservoir	Continuous	
Sex (I)	Participant sex	Individual	Individual risk factor	Binary	0 = female; 1 = male
Age (I)	Participant age	Individual	Individual risk factor	Continuous	
Education (I)	Highest level of schooling completed	Individual	Individual risk factor	Ordinal	1. None<br>2. Elementary School<br>3. Middle School or Higher
High-Risk Occupation (I)	High-risk occupation	Individual	Occupational risk factor	Categorical	0. Not farmer<br>1. Farmer or Fisher
Assets (H)	Number of household assets owned	Household	SES	Discrete	One point each is assigned for ownership of nine items including computers, televisions and trucks. One point for living in a brick or concrete home (vs. adobe)
Assets (V)	Mean number of assets owned per household	Village	SES	Continuous	
Well water (H)	Household uses well water	Household	WASH	Binary	Minor variations in questionnaire over time*
Well water (V)	Percent of households that use well water	Village	WASH	Continuous	
Improved sanitation (H)	Household has a biogas and/or 3-compartment toilet	Household	WASH	Binary	Minor variations in questionnaire over time†
Improved sanitation (V)	Percent of households with improved sanitation	Village	WASH	Continuous	
County (C)	County of residence	County	County	Binary	

SES: Socio-economic Status
WASH: Water, sanitation and hygiene

* In 2007, the survey directly asked if a household had a well ("well water"), but missing responses were relatively high (N=80). The 2007 survey also included questions as to whether people used well water for household activities such as cooking, washing, or bathing. These responses were used to create a derived "welluse" variable. Missing 2007 "well water" values were filled using the "welluse" variable, making 2007 data comparable with later years. From 2010 to 2019, the question was changed to "Does your household get water from a well?", which had fewer missing responses and was used to classify household well water use.
