# Supplementary figures and images for: One health at the last mile: Multi-scale predictors of Schistosoma japonicum infection in southwest China across two decades of control
Source: PLoS Negl Trop Dis. 2026 Feb 23;20(2):e0013573. doi: 10.1371/journal.pntd.0013573 (PMC12928498; doi:10.1371/journal.pntd.0013573)

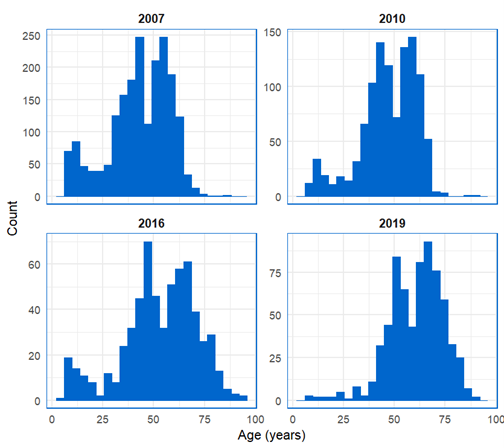

Supplement: S1 Fig — (PNG) [file pntd.0013573.s003.png]

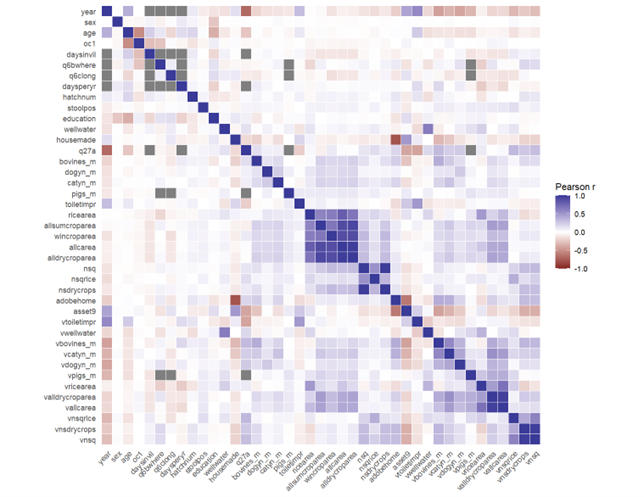

Supplement: S2 Fig — (PNG) [file pntd.0013573.s004.png]
