# Supplementary material for: One health at the last mile: Multi-scale predictors of Schistosoma japonicum infection in southwest China across two decades of control
Source: PLoS Negl Trop Dis. 2026 Feb 23;20(2):e0013573. doi: 10.1371/journal.pntd.0013573 (PMC12928498; doi:10.1371/journal.pntd.0013573)
Supplement: Attachment — Submitted filename: Response_to_reviewers_Jan2026.docx [file pntd.0013573.s006.docx]

We thank the reviewers for their thorough reading of our manuscript and their constructive, insightful comments. The suggestions on clarifying our interpretation, expanding methodological transparency for the GBM models (including hyperparameters, sensitivity analyses, and representativeness), and more clearly articulating site-specific limitations and model generalizability has helped us improve our manuscript. We have revised the introduction, methods, results, and discussion sections accordingly and believe the manuscript is stronger and more impactful as a result of this feedback.

**Methods**

**Reviewer #1: (No Response)**

**Reviewer #2: The study’s objectives are clearly articulated and aligned with its elimination-focused context, though the hypotheses could be more explicitly framed to reflect correlation-based rather than causal inference. The overall design, integrating multiscale (individual, household, and village) data, is appropriate and methodologically strong. The study population is well described and relevant to the research question.**

**The statistical approach (GBM) is suitable for exploring complex** **relationships, additional details on model hyperparameters, variable selection, and sensitivity analyses would improve transparency and rigor. The sample size appears adequate, though more discussion on representativeness and site-specific limitations would be helpful. There are no apparent ethical or regulatory concerns.**

Response: To address the reviewer’s comments about making it clear that our results are correlation-based rather than causal inference, we edited the methods, results and discussion to make it clear that our analysis is identifying predictors and associations. We also wrote in the limitations that although our work is a step toward casual inference, the modelling framework we used was not designed to estimate causal effects and draw attention to the limitations of our observational structure including unmeasured confounding, measurement error. We continue to frame our motivating questions in the introduction as causal questions since ultimately, we are interested in advancing our understanding of end-game schistosomiasis transmission dynamics. We have edited language to make clear that our approach uses a machine learning prediction framework.

In response to the reviewer’s suggestion, we have now included sensitivity analyses regarding the performance of our models using 20 bootstrapped iterations of the models. We included more details on hyperparameters and variable selection in the methods; this included the range of our hyperparameter grid search and additional lines in the methods section on how we addressed multi-collinearity.

We also included more discussion on representativeness and site-specific limitations in our methods and discussion section and performed sensitivity analyses to report model performance uncertainty.

**Reviewer #3: The methods are well written, and no revisions are necessary**

**Results**

**-Does the analysis presented match the analysis plan?**

**-Are the results clearly and completely presented?**

**-Are the figures (Tables, Images) of sufficient quality for clarity?**

***Reviewer #1: (No Response)***

**Reviewer #2: The analyses presented overall align with the study’s objectives and design. Results are clearly organized and correspond to the stated analytical framework, though additional transparency regarding GBM model parameters and sensitivity analyses would strengthen confidence in the findings. Figures and tables are of acceptable quality but could be improved by ensuring captions are fully interpretive, and that performance metrics include accompanying p-values or confidence intervals for clarity.**

Response: We revised most table and figure captions to be more comprehensible and support interpretation. We added text to the methods that provide greater detail regarding GBM model parameters including the range of hyperparameters we considered using grid search. We also added sensitivity analyses to evaluate model performance uncertainty. We are unable to provide accompanying p-values because our primary analytical approach used gradient boosted machines (GBMs), which do not yield p-values for individual predictors or performance metrics in the way that traditional regression models do. Because the goal of our analysis was prediction rather than formal hypothesis testing, we focused on estimating predictive performance and effect patterns with appropriate measures of uncertainty represented as our bootstrapped partial dependence plots rather than p-values.

***Reviewer #3: The results are well presented and no revisions are necessary***

**Conclusions**

**-Are the conclusions supported by the data presented?**

**-Are the limitations of analysis clearly described?**

**-Do the authors discuss how these data can be helpful to advance our understanding of the topic under study?**

**-Is public health relevance addressed?**

**Reviewer #1: (No Response)**

**Reviewer #2: The conclusions are overall supported by the data presented, although the discussion at times 'overinterprets' correlations as causal relationships. Reframing this or re-wording this a bit would improve the rigor of the paper. The limitations of the analysis, particularly the site-specific sampling and model constraints, should be discussed more explicitly. Strengthening the discussion on how findings inform surveillance and control strategies would enhance its public health relevance and broader impact.**

Response: We edited our discussion and conclusion to clarify that our analysis evaluated predictors of schistosomiasis infection and added text to the discussion that highlights distinctions between prediction and causal inference approaches. We added more sentences to our discussion and conclusion sections on the generalizability of our results, limitations of our site-specific sampling, and limitations regarding the use of gradient boosting machines. We also added an additional paragraph in the conclusion to address how the findings will inform surveillance and control strategies.

**Reviewer #3: Minor edits are suggested (see general comments)**

**Editorial and Data Presentation Modifications?**

**Use this section for editorial suggestions as well as relatively minor modifications of existing data that would enhance clarity. If the only modifications needed are minor and/or editorial, you may wish to recommend “Minor Revision” or “Accept”.**

**Reviewer #1: (No Response)**

**Reviewer #2: Acceptable**

**Reviewer #3: (No Response)**

**Summary and General Comments**

**Use this section to provide overall comments, discuss strengths/weaknesses of the study, novelty, significance, general execution and scholarship. You may also include additional comments for the author, including concerns about dual publication, research ethics, or publication ethics. If requesting major revision, please articulate the new experiments that are needed.**

**Reviewer #1: This research, titled "One Health at the Last Mile: Multi-scale Predictors of Schistosoma japonicum Infection in Southwest China across Two Decades of Control," employs a Gradient Boosting Machine (GBM) model to analyze the risk factors for schistosomiasis infection in Sichuan Province, China, during two critical periods: the re-emergence phase (2007–2010) and the near-elimination phase (2016–2019). By integrating multi-scale variables at the individual, household, and village levels—such as agricultural practices, animal hosts, socioeconomic status, and water sanitation facilities—the study reveals significant shifts in the relative importance of risk factors as control efforts progressed. It finds that in the near-elimination phase, household-level factors like agricultural activities and pet ownership became more influential, while the impact of village-level water sanitation facilities diminished. The study emphasizes the need for targeted intervention strategies during the elimination phase, focusing on high-risk households and agricultural practices.**

**Innovations:**

**Multi-scale analysis: The study combines variables at the individual, household, and village levels to comprehensively assess the risk of schistosomiasis transmission.**

**Temporal comparison: By comparing the re-emergence and near-elimination phases, it uncovers dynamic evolutionary patterns in risk factors.**

**One Health perspective: It integrates human, animal, and environmental factors, aligning with the "One Health" concept.**

**Policy implications: Provides empirical evidence for precision interventions and monitoring strategies during the elimination phase.**

Response: We thank the reviewer for this positive feedback.

**Some issues in the study require reconsideration:**

**Model generalization: The data were sourced from specific high-risk villages in Sichuan Province, and the number of infection cases in 2019 was extremely low (n=7). Did the authors consider validating the model’s generalizability through cross-validation or external datasets? How did they ensure the conclusions are applicable to other low-endemic areas?**

Response: We appreciate this important point regarding model generalizability and the small number of infections in 2019. Our primary objective was to explore how predictors of *S. japonicum* infection differed between a re-emergence period and a near-elimination period within these sentinel, high-risk villages. To evaluate generalizability and reduce overfitting, we used 5-fold cross-validation for our GBM models. For each time period, model performance metrics (AUC and other measures) were estimated across folds, and we additionally used bootstrapping to obtain confidence intervals, which reflect the higher uncertainty in 2019 due to the small number of cases. Because no external datasets with comparable village- and household-level data were available for formal external validation, we reframed our conclusions more cautiously and included your points as limitations in the discussion section.

We revised the discussion to explicitly state that the findings for 2019 should be interpreted as hypothesis-generating, and that generalization to other low-endemic areas should consider local ecology, control history, and surveillance capacity. We also wrote that several of the key predictors identified (e.g., age structure, sanitation, livestock ownership) are consistent with prior schistosomiasis literature, which supports the plausibility of our findings, while still acknowledging that external validation in other low-endemic settings is an important next step.

**Variable selection and collinearity: The study includes multiple agricultural and environmental variables, which may be highly correlated (e.g., crop area and fertilization methods). How did the authors address collinearity among variables? Did they assess the impact of collinearity on variable importance rankings?**

Response: We recognize that collinearity may influence variable importance rankings, as indicated by previous literature. To that extent, we avoided including intuitively correlated variables from the analysis, including areas of overlapping crop type and fertilization methods (e.g. we aggregated all non-rice crops into a single, “dry crop” variable). We also would have selected a single variable out of clusters of multicollinearities to a representative of similar variables, but none of the variables included in the final analysis were correlated to a high degree. We included additional lines in the methods section on how we addressed multi-collinearity and added a correlation matrix to the appendix.

**Seasonal bias: The infection survey in 2016 was conducted in summer, while other years were surveyed in winter. Could seasonal differences have affected infection detection results and model predictions? Did the authors attempt to adjust for or address this potential bias?**

Response: We appreciate this important point regarding potential seasonal bias. In our study setting, household and village questionnaires were administered during the summer (aligned with most agricultural activity, including rice cultivation), whereas infection testing was conducted in winter for most years but occurred in summer in 2016. Because schistosomiasis transmission and infection detectability can vary seasonally and because routine praziquantel treatment schedules may reduce observed prevalence later in the year differences in survey timing could have influenced measured infection outcomes and, in turn, model predictions for 2016. We did not have sufficient repeated within-year infection measurements to formally test for seasonality; therefore, we now explicitly acknowledge the 2016 summer testing as a limitation in the discussion.

**Mechanism of animal hosts: The study found that livestock rearing became more important in the elimination phase, but the mechanism remains unclear. The authors suggest that cats may reduce transmission by controlling rodents, while dogs may increase risk. Is there further data or analysis to support these hypotheses? Were animal infection detection data considered?**

Response: We appreciate the opportunity to address this point. Our dataset included detailed household-level information on ownership of bovines, dogs, and cats, but did not include animal infection testing for these other species during the study period. (This work was planned for summer 2020 but prevented by the pandemic). Therefore, our proposed mechanisms are based on ecological reasoning and prior literature, rather than direct measurement of animal infection in this cohort. We included literature in the discussion that provides evidence for the transmission risk of animal reservoirs including dogs and cats and acknowledge the uncertainty of our findings and that concurrent testing of humans and other animal hosts in this context would be ideal and is a major area of interest for future studies.

**Age-related risk shift: The high-risk age group shifted from 40–60 years to 80 years and older, which the authors attribute to the outflow of young rural populations. Is there direct data on population mobility or occupational exposure to support this explanation? Were behavioral pattern changes among the elderly considered?**

Response: We agree that the observed shift in age-specific risk is an important finding to unpack. Our study did not collect direct longitudinal migration histories or detailed occupational trajectories, so our explanation based on rural out-migration is inferential and based on existing literature on broader demographic trends in rural China rather than more specific behavioral pattern changes among the elderly. We collected data on the age of survey participants and have added histograms of age group distribution to the appendix. This also follows our prior analysis by Buchwald (2021) that showed out-migration from villages was associated with reduced water contact.

**Feasibility of intervention strategies: The authors recommend increasing targeted interventions for high-risk households during the elimination phase. How can these households be identified in practice? Is it feasible to implement such precision measures in resource-limited areas? Was a cost-effectiveness analysis conducted?**

Response: We thank the reviewer for these thoughtful comments. A feasibility and cost-effectiveness analysis was outside the scope of this analysis but is a valuable direction for future investigations. Our analysis shows it is possible to identify high-risk households using data that are commonly collected or can be obtained with some effort in elimination programs: annual/seasonal infection surveillance registries, brief household WASH/agricultural questionnaires, and village administrative records of crop practices and sanitation infrastructure (particularly for *S. japonicum).* A next step for future analyses would be to translate these predictors into an implementation-ready risk stratification tool and evaluate, from a program perspective, whether risk-based targeting reduces costs and staff burden while maintaining sensitivity compared with population-wide surveillance in low-prevalence settings.

**Data missingness and imputation: The study had 3.27% missing data, which was imputed using random forests. Was the impact of the imputation method on the stability of the results evaluated? Was a sensitivity analysis conducted to address potential biases introduced by missing data?**

Response: To assess whether imputation influenced model stability, we conducted a sensitivity analysis by refitting the full modeling pipeline across 20 bootstrapped iterations using different random seeds. Because most imputation methods include a stochastic component changing the random seed can yield slightly different imputed values, especially when missingness is non-trivial, so refitting the full pipeline across 20 bootstrap iterations with different seeds tests whether results are robust to imputation-induced variability.

We observed that model performance and the ranking of influential predictors were broadly consistent across iterations. We reported the model performance with 95% confidence intervals in figure 1. We edited table 1 to include 95% confidence intervals from our bootstrapping sensitivity analysis and added a line in the methods describing this.

**Reviewer #2: Overall a strong manuscript. Some issues, minor concerns are described in the section above already.**

**Reviewer #3: This is a well written, interesting and informative paper looking at the predictions of S. japonicum transmission at different stages of control and elimination. The analyses seems robust and the outcomes will be very useful for interventions going forward.**

**The introduction would benefit from more background to include**
**• The main human species – Africa and Asia and the global burden (not just China)**

Response: We added the main human species found in Africa and Asia to the introduction.

**• The key feature of transmission in China (zoonotic transmission)**

Response: We added that that transmission of *S. japonicum* is distinguished by the large number of definitive hosts, including humans and bovines.

**• More information on the different animal hosts and transmission environments**
**• The snails, environment and general life-cycle (how transmission happens)**
**• Clearly define S. japonicum as having bovines as reservoirs but that S. japonicum is zoonotic – although they are connected they have different definitions.**

Response: To address the above three points, we added more details to the introduction providing examples of different animal hosts, a brief sentence about the life-cycle of transmission and that *S. japonicum* is zoonotic not only through bovines.

**• Define elimination (e.g. as a public health problem or transmission interruption)**

Response: We defined elimination under WHO goals and included it in the introduction.

**Line 64 – The terminology should be intestinal schistosomiasis caused by S. japonicum.**

CSPH-M: We added that *S. japonicum* causes intestinal schistosomiasis.

**Line 123 – add detail as to what the animal reservoirs were**

Response: We expanded the animal reservoirs list in the introduction.

**Discussion**
**• It would be good to also mention the different behaviours of cats and dogs in terms of their involvement S. japonicum transmission. e.g. dogs like water and so are more likely to be exposed compared to cats.**

Response: We added how cat and dog behaviors may contribute to transmission in the discussion section.

**• It would be good to have a sentence or two on the boarder aspects of the work beyond Africa. How could this type of work support Schistosomiasis control and elimination on Brazil and Africa.**

Response: We added two sentences to the end of the discussion expanding on how our work is applicable to control efforts internationally.

**General – in various places the words “parasite” or “schistosome” are used whereas the authors should be more specific and use S. japonicum. Also, check that all species names are in Italics.**

Response: We replaced all ‘parasite’ and ‘schistosome’ terms used in the manuscript with the more specific *S. japonicum* and edited all species names to be in italics.
